# Association of Dietary and Lifestyle Inflammation Score With Cardiorespiratory Fitness

**DOI:** 10.3389/fnut.2022.730841

**Published:** 2022-03-30

**Authors:** Mena Farazi, Ahmad Jayedi, Zahra Noruzi, Nasim Janbozorgi, Kurosh Djafarian, Sakineh Shab-Bidar

**Affiliations:** ^1^Community Nutrition Department, School of Nutritional Sciences and Dietetics, Tehran University of Medical Sciences (TUMS), Tehran, Iran; ^2^Clinical Nutrition Department, School of Nutritional Sciences and Dietetics, Tehran University of Medical Sciences (TUMS), Tehran, Iran

**Keywords:** cardiorespiratory fitness, dietary and lifestyle inflammation score, diet, lifestyle, inflammation

## Abstract

**Objective:**

We aimed to assess the potential association of dietary (DIS) and lifestyle inflammation score (LIS) and their joint association (DLIS) with cardiorespiratory fitness (CRF) in Tehranian adults.

**Design:**

The present study was designed cross-sectional.

**Participants:**

A total of 265 males and females aged 18–70 years (mean ± SD: 36.9 ± 13.3) were entered in the present cross-sectional study. Eligible participants were healthy men and women who were free of medications and had no acute or chronic infection or inflammatory disease.

**Measures:**

The DIS was calculated by the use of data from 18 anti- and pro-inflammatory dietary components, and the LIS by three non-dietary components including physical activity, smoking status, and general adiposity, with higher scores indicating a more pro-inflammatory diet and lifestyle, respectively. The DLIS was calculated by summing the DIS and LIS. CRF was assessed by the Bruce protocol and VO_2_ max was measuredas the main variable of CRF. The odds ratio (OR) and 95% confidence interval (CI) of CRF across tertiles of the DIS, LIS, and DLIS were estimated by logistic regression analysis with considering age, gender, energy intake, marital and education status, and occupation as confounders.

**Results:**

The DLIS ranged from −2.10 to 0.38 (mean ± SD: −1.25 ± 0.64). In the model that controlled for all variables, the ORs of CRF for the second and third tertiles of the DLIS as compared to the first tertile were 0.42 (95%CI: 0.20, 0.90) and 0.12 (95%CI: 0.05, 0.32), respectively (P-trend < 0.001). There was a strong inverse association between the LIS and CRF (OR_thirdvs.firsttertile_: 0.12, 95%CI: 0.05, 0.32). There was no association between DIS and CRF.

**Conclusion:**

The present study examined the joint association of inflammation-related lifestyle behaviors with CRF and found a strong inverse association between a pro-inflammatory lifestyle with CRF. We did not find any association between dietary inflammatory properties with CRF. Future studies should address the relationship between the inflammatory potential of the diet and CRF.

## Highlighting Key Findings

Strong inverse associated was found between inflammatory lifestyle and CRF. No association was found between dietary inflammatory index and CRF. No association was found between CRF and DLIS.

## Introduction

Cardiorespiratory fitness (CRF) is applied to evaluate a person's ability to perform physical work, and its definition is maximum capacity of the cardiovascular and respiratory system to supply oxygen to the skeletal muscles through exercise (1). CRF represents the ability to transport inhaled oxygen to the mitochondria (2). A maximal cardiorespiratory exercise test is performed to measure CRF and accordingly, it is defined as the level of oxygen consumption at peak exercise performance (VO_2_ peak), or the maximal oxygen consumption (VO_2_ max), so VO_2_ peak or VO_2_ max is the main variable of CRF (2). CRF is an important health indicator because of its strong relationship with all-cause mortality (3). It is thought to be more important than other traditional risk factors such as smoking, hypertension, high serum cholesterol, and type 2 diabetes (2). CRF can be useful in predicting the risk of cardiovascular disease (CVD), systematic arterial hypertension, diabetes, metabolic syndrome, and cancer (4–9). It has been shown that CVD death rates in individuals with moderate to high levels of CRF were 71% lower than those who are unfit (10). CRF was inversely associated with circulating pro-inflammatory factors including C-reactive protein (CRP), Interleukin (IL)-6, and IL-18, and positively associated with the anti-inflammatory cytokine IL-10 (11). Recently, many studies have reported that high CRF is associated with lower levels of CRP (12, 13).

Although nearly 50% of the individual difference in CRF is thought to be determined by genetic factors, it can also be influenced by lifestyle-related factors such as physical activity, smoking, and body mass index (BMI) (14–16). A lifestyle that includes physical activity can improve the level of CRF especially in those who have a low level of CRF (16). The World Health Organization (WHO) has reported that the recommended physical activity (PA), defined as at least 30 min/d moderate PA, is not met by more than 60% of the world's population (17).

Meanwhile, cigarette smoking has unfavorable effects on CRF, in a way that, aerobic capacity and, thus, the ability of oxygen supply during exercise, declines substantially in smokers (8). Non-smokers had a higher level of VO_2_ max in comparison with current smokers and quit smokers (14). Increased BMI was also related to lower CRF (15). Poor dietary choices and a massive decrease in work-related PA in both gender and household women lead to the global prevalence of obesity during the past decades (18–20).

The relationship between CRF and dietary intake in young and older adults have been examined by some investigations. It has been documented that older adults who have higher CRF are more likely to follow the dietary recommendations than those who are unfit (21, 22). There is evidence that the diet-disease association may be mediated partly by the potential impact of dietary habits on CRF (23, 24).

Low CRF is an unfavorable health condition that is highly accompanied by low-grade systemic inflammation (25). Sedentary lifestyle (26), cigarette smoking (27), adiposity (28), and poor diet quality (29) are also important drivers of systemic inflammation in the human body. Therefore, the effects of dietary and lifestyle-related factors on CRF may be mediated, in part, by their unfavorable impacts on low-grade systemic inflammation. However, the combined effects of these factors on CRF has not been investigated.

To estimate the inflammatory potential of dietary and lifestyle behaviors, a new index has been developed to take important anti- and pro-inflammatory dietary and non-dietary components into account. The dietary and lifestyle inflammation index (DLIS) is a new-developed index (30) that combined the effects of anti- and pro-inflammatory dietary components, as well as four lifestyle-related components including PA, alcohol drinking, cigarette smoking, and BMI to present a broad picture of the effect of human lifestyle on inflammation. Thus, considering the vital role of CRF in health, the objective of this cross-sectional study was to determine the association of DLIS with CRF in adults.

## Methods

### Study Population

In this cross-sectional study, 270 adult men and women with an age range from 18 to 70 years who lived in Tehran were recruited. Eligible individuals were invited to attend the School of [double blinded] through advertisement in the local media. Eligible subjects were selected from volunteers according to pre-specified inclusion criteria by using a convenience sampling method. Eligible participants were healthy men and women, aged between 18 and 70 years, who were free of medications and had no acute or chronic infection or inflammatory disease. Subjects were excluded if they used any supplementation, or were lactating or pregnant at the time of the study. All participants received information concerning the background and procedures of the study. Written informed consent was obtained from each participant before data collection. The ethical committee [double blinded].

### Demographic Factors

Trained interviewers recorded data about age, sex (male, female), education (Having or not having university education), marriage (single or married), smoking (never or former smoker, current smoker), and occupation (employee, housekeeper, retired, unemployed) by using pre-specified data extraction forms.

### Physical Activity

The generally validated International Physical Activity Questionnaire (IPAQ) was applied to evaluate PA levels. PA levels were expressed as metabolic equivalent minutes per week (MET-min/week) (31), and accordingly, subjects were classified into two groups as follow: no or low PA (<600 MET-minute/week) and moderate and high low PA (>600 MET-minute/week).

### Anthropometric Measurements

Anthropometric variables consisting of weight and height were measured by trained dieticians. Height was measured using a wall stadiometer (Seca, Germany) and recorded to the nearest 0.1 cm. Weight was measured by an adult's digital scales (808 Seca, Germany) with a sensitivity of 0.1 kg. Anthropometric measurements were performed barefoot and in light clothing. BMI was calculated as weight in kilograms divided by the square of height in meters.

### Dietary Assessment

A reliable and validated food frequency questionnaire (FFQ) with 168 food items which was common in Iran (32), was applied to measure usual dietary intakes. Trained nutritionists through face-to-face interviews have asked the frequency (on a daily, weekly, monthly, and annual basis) and amount of consumption of each food item during the past year from the participant. Dietary intakes were then converted to g/d according to household measures (33). Finally, the daily intake of energy and nutrients was estimated using Nutritionist IV software based on the US Department of Agriculture food composition database modified for Iranian foods (34).

### Calculating the Dietary and Lifestyle Inflammation Score

The method presented by Byrd et al. was used to calculate the DLIS in the present study (30) (Supplementary Table 1). The construction of this method was validated by a previous study. This score includes dietary inflammation score (DIS) and lifestyle inflammation score (LIS). Components of the diet include 19 variables and components of the lifestyle include four variables. The validation study reported that these dietary and non-dietary components have a significant effect on circulating concentrations of some pro-inflammatory biomarkers including IL-6, IL-8, and CRP or anti-inflammatory biomarkers such as IL-10. Then, the inflammatory potential of each component was scored according to whether it increased pro-inflammatory or declined anti-inflammatory markers, or it reduced pro-inflammatory or increased anti-inflammatory factors.

The DIS components include leafy greens and cruciferous vegetables, tomatoes, apples and berries, deep yellow or orange vegetables and fruit, other fruits and real fruit juices, other vegetables, legumes, fish, poultry, red and organ meats, processed meats, added sugars, high-fat dairy, low-fat dairy, coffee and tea, nuts, other fats, refined grains, starchy vegetables, and supplement score. All of these components were used other than supplement score due to the lack of information regarding supplement use in the study participants. The LIS components include smoking status (“former/never” or “current”), physical activity (“high or moderate” and “low or no physical activity”), and BMI (kg/m^2^) [“overweight (25–29.9)” and “obese (≥30)]” and alcohol intake. Alcohol intake was not included in the score because of the lack of information regarding the intake of alcohol in Iranian culture. In this study, the DLIS for each subject was calculated by summing the DIS and LIS. Higher DLIS (more positive) presents a more pro-inflammatory diet and lifestyle.

### Assessment of Cardiorespiratory Fitness

The maximum rate of oxygen consumed (VO_2_ max) was measured using a treadmill and respiratory gas analyzer (Cortex Metabolizer 3B) as the main variable of CRF. The subjects warmed up for 5 min on the treadmill at a speed of 5 km/h and then, the Bruce test was used to determine the VO_2_ max using standard procedures (35). After completing the Bruce test, the subjects walked at a speed of 4 km/h to cool down for 3 min and were encouraged to perform 5 to 10 min of stretching. The conditions for test cessation were: the heart rate up to 90% of the maximum heart rate, a respiratory exchange ratio over 1.1, and having a plateau in oxygen intake, despite increases in exercise intensity. The CRF was expressed as VO_2_ max and those in the above-median category (>32 vs. <32) were considered to have CRF.

### Statistical Analysis

The mean and standard deviation (SD) of continuous variables across tertiles of the DLIS were compared by one-way ANOVA test. The frequency of categorical variables across tertile of the DLIS was assessed by the chi-square test. The odds ratios (OR) and 95% confidence intervals (CI) of CRF (the above-median group as compared with the below-median group) across tertiles of the LIS, DIL, and DLIS and P-trend were determined through binary logistic regression analysis in the crude and adjusted models. In the first model, we adjusted for age, gender, and energy intake. Further adjustments were made for marital status, education, and occupation in the second model. Participants in the first quartile of the DLIS were considered as the reference group. The Statistical Package for the Social Sciences (SPSS version 26; SPSS Inc) was used for performing all statistical analyses. The statistical significance level was defined as *p* <0.05.

## Results

### General and Body Composition Characteristics

Subject characteristics within each tertile of the DLIS are shown in Table 1. The mean age of participants was 36.9 ± 13.3 years. Those in the top tertile were older and had heavier weight as compared to the first tertile. The mean BMI was 25.7 ± 4.7 kg/m^2^ that increased proportionally from the first to the third tertile (*p* < 0.001). The ranges of the DLIS and VO_2_ max in the study participants were from −2.10 to 0.38 (mean ± SD: −1.25 ± 0.64) and 17 to 54 (mean ± SD: 31.40 ± 7.51), respectively. The mean of VO_2_ max significantly declined across tertiles of the DLIS (*p* < 0.001). The DLIS was significantly related to marital status, occupation, PA (P for all < 0.001), and education status (*P* = 0.005), but was not related to current smoking (*P* = 0.09).

**Table 1:** Characteristics of the study participants across tertiles of the DLIS (*n* = 265).

Variables*	**T1 (*n* = 80)**	**T2 (*n* = 99)**	**T3 (*n* = 86)**	*P*-value**
DLIS range	(−2.10 to −1.87)	(−1.69 to −0.98)	(−0.80 to 0.38)	
Age (year)	32.8 ± 12.3	34.7 ± 11.8	42.8 ± 13.8	<0.001
Weight (kg)	62.5 ± 11.1	69.8 ± 12.2	85.8 ± 15.8	<0.001
BMI (kg/m^2^)	22.0 ± 2.50	24.5 ± 3.09	30.2 ± 4.11	<0.001
VO_2_ max	34.3 ± 7.94	32.1 ± 7.09	27.7 ± 6.17	<0.001
Gender (% men)	26.3%	36.4%	37.3%	0.26
Marital status (% married)	18.6%	36.4%	45%	<0.001
Current smoker (%)	38.4%	20.1%	41.4%	0.09
Occupation (%)				<0.001
Employee	31.9%	39.7%	28.4%	
Housekeeper	9.5%	33.3%	57.1%	
Retired	27.3%	9.1%	63.6%	
Unemployed	41.7%	45.0%	13.3%	
Physical activity (%)				<0.001
Low	0%	44.3%	55.7%	
Moderate (1–3 times/wk)	48.7%	32.6%	17.7%	
High (≥4 times/wk)	45.5%	32.7%	21.8%	
Education (%)				0.005
Diploma	22.9%	25.0%	52.1%	
Under diploma	15.0%	35.0%	50.0%	

*BMI, body mass index; DLIS, dietary and lifestyle inflammation score; T, tertile.*

*Data are presented as n (%) for categorical variables or mean ± standard deviation for continuous variables.

**The one-way analysis of variance and the chi-square test was used for comparison of continuous and categorical variables among tertiles of DLIS, respectively. P <0.05 was considered significant.

### Dietary Characteristics

Dietary intakes of the study participants among tertiles of the DLIS are reported in Table 2. Subjects in the highest tertile of the DLIS showed a higher intake of energy, carbohydrate, protein, fiber, leafy greens and cruiferous vegtables, apples and berries, tomatoes, other fruits and juices, other vegtables, reg and organ meat, high-far dairy, coffe and teanuts and refined grains and starchy vegtables (*P* <0.001). Participants in the highest tertile of the DLIS had higher consumption of added sugars. The intake of other dietary variables did not differ significantly across tertiles of the DLIS.

**Table 2:** Dietary intakes of study participants across tertile of the DLIS.

**Variables**	**T1 (*n* = 80)**	**T2 (*n* = 99)**	**T3 (*n* = 86)**	***P*-value**
DLIS range	(−2.10, −1.87)	(−1.69, −0.98)	(−0.80, 0.38)	
*Energy (kcal/d)	2,126 ± 651	2,297 ± 743	2,540 ± 838	<0.001**
*Carbohydrate (g/d)	294 ± 98.0	332 ± 118	346 ± 123	<0.001**
*Protein (gr/d)	81.9 ± 31.3	85.8 ± 32.6	100 ± 48.2	0.002**
*Total fat (g/d)	74.5 ± 29.6	75.3 ± 31.9	81.7 ± 35.4	0.15**
*Fiber (g/d)	14.4 ± 5.91	15.2 ± 6.54	17.7 ± 7.23	<0.001**
^a^PUFA (g/d)	15.8(10.5, 21.6)	14.6(9.10, 19.9)	13.0(8.71, 19.4)	0.11^b^
^a^MUFA (g/d)	22.7(15.9, 31.0)	20.1(15.1, 26.5)	20.3(13.9, 28.2)	0.15^b^
^a^Leafy greens and cruciferous vegetables (g/d)	26.9(16.9, 37.5)	19.1(10.1, 28.5 )	18.7(11.4, 26.6)	0.005^b^
^a^Tomatoes (g/d)	29.8(10.3, 41.5)	20.7)9.68, 31.1)	15.7(5.01, 20.7)	**<0.001** ^**b**^
^a^Apples and berries (g/d)	31.2(21.1, 49.2)	16.6(9.42, 28.4)	13.4(7.01, 20.3)	**<0.001** ^**b**^
^a^Deep yellow or orange vegetables and fruit (g/d)	42.6(28.9, 72.7)	23.5(13.3, 41.4 )	18.6(10.7, 24.2)	**0.14** ^**b**^
^a^Other fruits and fruit juices (100%) (g/d)	234(140, 325)	142(85.9, 203)	120(82.1, 178)	**<0.001** ^**b**^
^a^Other vegetables (g/d)	73.2(52.9, 110)	49.5(26.4, 72.4)	41.9(31.1, 70.6)	**<0.001** ^**b**^
^a^Legumes (g/d)	53.1(35.8, 85.5)	45.0(26.4, 65.7)	32.9(17.6, 77.8)	0.94^b^
^a^Fish (g/d)	0.01(0.0, 0.37)	0.12(0.0, 0.25)	0.0(0.0, 0.25)	0.77^b^
^a^Poultry (g/d)	0.33(0.06, 1.35)	0.26(0.6, 1.02)	0.16(0.02, 0.41)	0.13^b^
^a^Red and organ meats (g/d)	239(89.5, 358)	186(105, 334)	185(100, 307)	**<0.001** ^**b**^
^a^Processed meats (g/d)	23.7(15.2, 32.2)	23.4(15.2, 38.2)	23.0(15.4, 38.4)	0.16^b^
^a^Added sugars (g/d)	503(307, 745)	606(363, 824)	846(490, 1,151)	**0.014** ^**b**^
^a^High-fat dairy (g/d)	179(143, 286)	144(101, 239)	174(107, 266)	**<0.001** ^**b**^
^a^Low-fat dairy (g/d)	17.1(3.14, 30.0)	8.57(1.37, 30.0)	8.98(0.0, 30.0)	0.38^b^
^a^Coffee and tea (g/d)	3.83(0.89, 13.7)	3.66(0.62, 6.30)	2.46(0.30, 4.00)	0.009^b^
^a^Nuts (g/d)	17.2(7.44, 35.1)	13.2(7.13, 22.1)	10.3(5.34, 15.1)	0.01^b^
^a^Other fats (g/d)	15.3(6.61, 29.3)	12.4(5.29, 19.1)	10.8(3.69, 21.5)	0.26^b^
*Refined grains and starchy vegetables (g/d)	446 ± 182	512 ± 234	580 ± 298	0.001**

*DLIS, dietary and lifestyle inflammation score; T, tertile; PUFA, polyunsaturated fatty acid; MUFA, monounsaturated fatty acid.*

*Data are mean ± standard deviation for normal variable.

*^a^Data are median (interquartile range) for skewed variables.*

**Using Analysis of One-Way ANOVA. P < 0.05 was considered significant.

*^b^Using Analysis of Kruskal-Wallis Test. P < 0.05 was considered significant. Significant P-values were bolded*.

### Association Between DLIS With CRF

The ORs and 95%CIs of CRF among the tertiles of the DLIS, DIS, and LIS are shown in Table 3. The results showed a strong inverse association between higher DLIS and odds of CRF. In the crude model, there was no significant association between the second tertile of the DLIS and odds of CRF, but the third tertile was significantly and strongly associated with lower odds (OR: 0.28, 95%CI: 0.12, 0.44; *P* for trend < 0.001). The associations became much stronger when we controlled for confounding variables including age, sex, energy intake, marital status, education status, and occupation. In the fully adjusted model, the OR and 95%CI of the CRF for the second and third tertiles of the DLIS were 0.42 (95%CI: 0.20, 0.90) and 0.12 (95%CI: 0.04, 0.32), respectively (*P* for trend < 0.001).

**Table 3:** The association between the DLIS and cardiorespiratory fitness in adults (odds ratio and 95% confidence interval).

	**Categories of the DLIS**			
**CRF (No. of participants)**	**T1 (*n* = 80)**	**T2 (*n* = 99)**	**T3 (*n* = 86)**	*P for trend
DLIS range	(−2.10, −1.87)	(−1.69, −0.98)	(−0.80, 0.38)	
Crude	1.0	0.58 (0.31–1.06)	0.28 (0.12–0.44)	<0.001
Model 1	1.0	0.43 (0.21–0.89)	0.10 (0.04–0.25)	<0.001
Model 2	1.0	0.42 (0.20–0.90)	0.12 (0.04–0.32)	<0.001
	**Categories of the DIS**			
DIS range	(−3.02, −0.93)	(−0.92, −0.05)	(−0.04, 1.55)	
Crude	1.0	0.88 (0.49–1.57)	1.20 (0.66–2.18)	0.54
Model 1	1.0	0.98 (0.46–2.07)	1.23 (0.46–3.32)	0.69
Model 2	1.0	1.07 (0.48–2.38)	1.07 (0.38–3.32)	0.89
	**Categories of the LIS**			
LIS range	(−0.41, −0.18)	(0.00, 0.71)	(0.89, 2.07)	
Crude	1.0	0.58 (0.31–1.06)	0.23 (0.12–0.44)	<0.001
Model 1	1.0	0.43 (0.21–0.89)	0.10 (0.04–0.25)	<0.001
Model 2	1.0	0.42 (0.19–0.89)	0.12 (0.05–0.32)	<0.001

*CRF, cardiorespiratory fitness; DLIS, dietary and lifestyle inflammation score; DIS, dietary inflammation score; LIS, lifestyle inflammation score; T, tertile.*

*P-trend is obtained by logistic regression analysis.

*Model 1: adjusted for age, gender, and energy intake. Model 2: additionally, adjusted for marital status, education status, and occupation*.

The OR of CRF among the tertiles of DIS showed that there was no significant association between DIS and CRF. This result remained stable after controlling for confounders (OR: 1.07, 95%CI: 0.37, 3.05, *P* for trend 0.89). Table 3 presents a strong association between LIS and CRF, in a way that odds of having CRF decreased proportionally from the first to the third tertile of LIS in the crude model. Adjustment for confounders made this result stronger (OR_thirdvs.firsttertile_: 0.12, 95%CI: 0.05, 0.32, *P* for trend < 0.001).

## Discussion

To the best of our knowledge, this is the first study that assessed the association of the inflammatory potential of the diet and lifestyle combined with CRF in young adults. The main finding of our study was that higher adherence to a pro-inflammatory diet and lifestyle, reflected by high DLIS, had a strong inverse association with odds of having CRF. There was also a strong inverse association between LIS and CRF. However, our results indicated no association between the inflammatory potential of the diet, as assessed by DIS, with CRF.

In this study, participants were more likely to have a lower level of CRF with a pro-inflammatory lifestyle. The association between single components of the DLIS and levels of CRF has been investigated in previous research. Recent investigations from the Aerobics Center Longitudinal Study in the US presented that adopting a more anti-inflammatory lifestyle including having higher PA, being at a normal weight, and not smoking were related to higher levels of CRF in both men and women (36). Existing evidence suggests an inverse association between cigarette smoking and levels of CRF (37). De Borba et al. reported that non-smokers showed a higher level of VO_2_ max compared to active smokers and passive smokers. However, the VO_2_ max of passive smokers did not differ from active smokers (14). An experimental investigation indicated that even a short-time passive smoker exposure in non-smoking adults can exert substantial adverse effects on the cardiorespiratory and immune response to PA (38).

A randomized controlled trial demonstrated evidence for a dose-response association between PA and progress in CRF. The study mentioned that promoting either quality or quantity of PA seems to have extra influence on CRF (39). Chatrath et al. showed that overweight children are more likely to perform poorly physical tests than their non-obese peers (40); however, such a result was not found in boys (15).

There is also evidence for an association between dietary factors and CRF. Carbone et al. reported that carbohydrate intake, especially in the form of sugars, was inversely, and higher consumption of mono- and polyunsaturated fats were positive, associated with CRF (41). A cross-sectional evaluation within the CARDIA study presented that there is a positive association between diet quality, as assessed by a pre-defined diet quality index, and CRF. The study also showed a negative association between a data-driven meat pattern, rich in red and processed meat, and CRF, and in contrast, a positive association for fruit and vegetable patterns (42). Borney et al. demonstrated that lower consumption of carbohydrates, fiber, folate, calcium, vitamin A, vitamin B-6, and vitamin C; and higher consumption of total, saturated and monounsaturated fats and cholesterol were associated with low physical fitness in both genders (21). However, similar to our findings, some investigations have reported no association between dietary habits and CRF (43–46). The present study is the first try to examine the association of inflammatory potential of the diet with CRF and this highlights the need for further research to fully investigate the association between DIS and CRF.

Recent investigations presented that there is an association between diet and lifestyle with inflammatory factors (47). Montonen et al. documented that there was a relationship between high consumption of whole-grain bread and lower levels of high-sensitive C-reactive protein, whereas a high intake of red meat was related to higher levels (48). Dietary patterns characterized by high intakes of refined starches, sugar, and saturated and trans-fatty acids and low intakes of natural antioxidants, nuts, fiber (from fruits and vegetables), and whole grains may lead to a stimulation of the innate immune system, most likely by an extreme creation of pro-inflammatory cytokines related to a declined production of anti-inflammatory cytokines. Choosing healthy sources of carbohydrate, fat, and protein, in association with regular PA and not smoking have a vital role against inflammatory biomarkers (49–51). Lifestyle factors such as smoking cessation, augmenting PA, and weight loss are associated with a reduction in CRP concentration (52, 53). Therefore, CRF may be associated with inflammatory factors (54, 55), and as a result, the strong inverse association between the DLIS and levels of CRF found in the present study may be mediated partly by inflammatory pathways.

Strengths of this study that are worth to be mentioned are the careful measurements of dietary intakes of each participant which were recorded by skilled nutritionists and valid and reliable questionnaires. Also, we applied a valid tool to calculate the DLIS. Potential limitations should also be considered. The results of our investigation ought to be taken with caution due to its cross-sectional design. Hence, prospective studies are needed to confirm the long-term influence of the DLIS on CRF. Moreover, in this study two items were not included for calculating the DLIS. Missing items include supplementation score and alcohol intake. Although we adjusted several confounding variables in our study, the results may have been affected by residual confounding. Finally, we used FFQ for dietary assessment that has been shown to have some limitations in evaluating dietary information (56). Also, we have to mentioned some of variables were skewed.

## Conclusions and Implications

In conclusion, the present study examined the joint association of inflammation-related lifestyle behaviors including cigarette smoking, sedentary lifestyle, and being overweight/obese with CRF and found a strong inverse association between a pro-inflammatory lifestyle with CRF. We did not find any association between dietary inflammatory properties with CRF. Future studies should address the relationship between the inflammatory potential of the diet and CRF.

## Data Availability Statement

The raw data supporting the conclusions of this article will be made available by the authors, without undue reservation.

## Ethics Statement

The studies involving human participants were reviewed and approved by the Ethical Committee of the Tehran University of Medical Sciences approved the study protocol (Ethical Approval ID: IR.TUMS.VCR.REC.1397.472). The patients/participants provided their written informed consent to participate in this study.

## Author Contributions

SS-B and KD contributed to the conception/design of the research. ZN and NJ contributed to the acquisition of data. MF and ZN participated in the analysis and interpretation of the data. MF drafted the manuscript. AJ critically revised the manuscript. SS-B agrees to be fully accountable for ensuring the integrity and accuracy of the work. All authors read and approved the final manuscript.

## Funding

This manuscript has been granted by Tehran University of Medical Sciences (Grant No: 33887). The funder had no role in the design, analysis or writing of this article.

## Conflict of Interest

The authors declare that the research was conducted in the absence of any commercial or financial relationships that could be construed as a potential conflict of interest.

## Publisher's Note

All claims expressed in this article are solely those of the authors and do not necessarily represent those of their affiliated organizations, or those of the publisher, the editors and the reviewers. Any product that may be evaluated in this article, or claim that may be made by its manufacturer, is not guaranteed or endorsed by the publisher.
