# Supplementary material for: Association of Dietary and Lifestyle Inflammation Score With Cardiorespiratory Fitness
Source: Front Nutr. 2022 Mar 30;9:730841. doi: 10.3389/fnut.2022.730841 (PMC9005778; doi:10.3389/fnut.2022.730841)
Supplement: Supplementary file 1 [file Table_1.pdf]

Supplementary Table 1. Components of the DIS and LIS, rationales for inclusion, and assigned weights

Components	Rationales for inclusion	Weights
**DIS components**		
Leafy greens and cruciferous vegetables	Kale, spinach, lettuce (iceberg, head, romaine, or leaf), broccoli, Brussels sprouts, cabbage, cauliflower, parsley, watercress	−0.14
Tomatoes	Tomatoes, tomato juice, tomato sauce, salsa	−0.78
Apples and berries	Fresh apples, pears, apple juice or cider, strawberries, blueberries, raspberries, cherries	−0.65
Deep yellow or orange vegetables and fruit	Cantaloupe, peaches, carrots, dark yellow or orange squash, figs	−0.57
Other fruits and real fruit juices	Fresh fruits other than those listed above (e.g., pineapples, honeydew, grapes, kiwi, watermelon, lemon, grapefruit, and oranges), orange juice, grapefruit juice, grape juice, and other real fruit juice	−0.16
Other vegetables	Vegetables other than those listed above (e.g., okra, green peppers, onions, zucchini, and eggplant)	−0.16
Legumes	String beans, peas, lima beans, lentils, and other beans (excluding soybeans)	−0.04
Fish	Tuna fish, salmon, other light and dark meat fish, breaded fish cakes or fish sticks	−0.08
Poultry	Chicken or turkey with and without skin	−0.45
Red and organ meats	Hamburger, beef, pork, lamb, liver, gizzards, other organ meats	0.02
Processed meats	Bacon, beef or pork hotdogs, chicken or turkey hot dogs, salami, bologna, other processed meats	0.68
Added sugars	Sugar-sweetened soda, punch, lemonade, chocolate candy bars, other mixed candy bars, candy without chocolate, jams, jellies, preserves, syrup or honey, dried or canned fruit	0.56
High-fat dairy	Whole milk, 2% milk, cream, high-fat ice cream, high-fat yogurt, cream cheese, other high-fat cheeses	−0.14
Low-fat dairy	Skim milk, 1% milk, low-fat yogurt, low-fat ice cream, low-fat cottage or ricotta cheese, low-fat cheeses	−0.12
Coffee and tea	Coffee (decaffeinated and regular), herbal and non-herbal tea	−0.25
Nuts	Peanut butter, peanuts, other nuts	−0.44
Other fats	Mayonnaise, margarine, butter, vegetable oil	0.31
Refined grains and starchy vegetables	Cold and cooked breakfast cereal, white or dark bread, bagels, English muffins, rolls, corn bread, white rice, pasta, pancakes, waffles, potatoes (French fried, scalloped, baked, boiled or mashed), sweet potato/yams, potato chips, crackers, tortillas, popcorn, pretzels, cookies, brownies, doughnuts, cake, pie, sweet rolls, coffee cakes, granola bars	0.72
**LIS components**		
Moderately physically active[1]	Exercises 1–3 times/wk vs. does not exercise	−0.18

Heavily physically active[2]	Exercises ≥ 4 times/week	−0.41
Current smoker[3]	Currently smokes tobacco vs. does not currently smoke tobacco	0.50
Overweight BMI[4]	Overweight BMI	0.89
Obese BMI[5]	Obese BMI	1.57

Abbreviations: DIS, dietary inflammation score; LIS, and lifestyle inflammation score

[1] low or no physical activity

[2] high or moderate

[3] former/never." or "current

[4] overweight (25–29.9)

[5] obese (≥30)
